# Innovative procedure for measuring left ventricular ejection fraction from ^18^F-FDG first-pass ultra-sensitive digital PET/CT images: evaluation with an anthropomorphic heart phantom

**DOI:** 10.1186/s40658-021-00387-2

**Published:** 2021-05-20

**Authors:** Emilie Verrecchia-Ramos, Olivier Morel, Paul Retif, Sinan Ben Mahmoud

**Affiliations:** 1 Medical Physics Unit, CHR Metz-Thionville, Mercy Hospital, 1 allée du château, 57530 Ars-Laquenexy, Metz, France; 2 Nuclear Medicine Department, CHR Metz-Thionville, Mercy Hospital, 1 allée du château, 57530 Ars-Laquenexy, Metz, France; 3 CNRS, CRAN, University of Lorraine, F-54000 Nancy, France

**Keywords:** LVEF, Digital PET/CT, “Pseudo-planar” PET, Heart phantom, First-pass

## Abstract

**Background:**

Left ventricular ejection fraction (LVEF) is usually measured by cine-cardiac magnetic resonance imaging (MRI), planar and single-photon emission-computerized tomography (SPECT) equilibrium radionuclide angiocardiography (ERNA), and echocardiography. It would be clinically useful to measure LVEF from first-pass positron-emission tomography/computed tomography (PET/CT) radionuclide angiography, but this approach has been limited by fast radiotracer diffusion. Ultra-sensitive digital PET systems can produce high-quality images within 3-s acquisition times. This study determined whether digital PET/CT accurately measured LVEF in an anthropomorphic heart phantom under conditions mimicking radiotracer first-pass into the cardiac cavities.

**Methods:**

Heart phantoms in end-diastole and end-systole were 3D-printed from a patient’s MRI dataset. Reference left ventricle end-diastole volume (EDV), end-systole volume (ESV), and LVEF were determined by phantom weights before/after water filling. PET/CT (3-s acquisitions), MRI, and planar and SPECT ERNA were performed. EDV, ESV, and/or LVEF were measured by manual and automated cardiac cavity delineation, using clinical segmentation softwares. LVEF was also measured from PET images converted to 2D “pseudo-planar” images along the short axis and horizontal long axis. LVEF was also calculated for planar ERNA images. All LVEF, ESV and EDV values were compared to the reference values assessed by weighing.

**Results:**

Manually calculated 3D-PET-CT-based EDV, ESV, and LVEF were close to MRI and reference values. Automated calculations on the 3D-PET-CT dataset were unreliable, suggesting that the SPECT-based tool used for this calculation is not well adapted for PET acquisitions. Manual and automated LVEF estimations from “pseudo-planar” PET images were very close/identical to MRI and reference values.

**Conclusions:**

First-pass “pseudo-planar” PET may be a promising method for estimating LVEF, easy to use in clinical practice. Processing 3D PET images is also a valid method but to date suffers from a lack of well-suited software for automated LV segmentation.

## Background

An important measure of the efficiency of myocardial pumping is left ventricular ejection fraction (LVEF), defined as the volumetric fraction of blood ejected from the left ventricle with each heartbeat. To date, the most reliable methods for LVEF assessment are cardiac cine magnetic resonance (MR) imaging [1] and ECG-gated planar or single-photon emission computed tomography (SPECT) equilibrium radionuclide angiocardiography (ERNA) [2, 3]. Echocardiography and ECG-gated myocardial perfusion scintigraphy can also be used to assess LVEF with less accuracy [4–7]. All of these methods have drawbacks [8]. MR imaging takes a long time, is more rarely available, and has many contraindications. Planar or SPECT ERNA involves ionizing radiation and low spatial resolution. Echocardiography accuracy is dependent on both patient and operator [9]. Finally, ECG-gated myocardial perfusion scintigraphy visualizes the myocardial wall rather than the cardiac cavities [10], involving systematic underestimation of LVEF.

An alternative to these LVEF-assessing methods is first-pass radionuclide angiography [11] with positron-emission tomography (PET). Cardiac ECG-gated PET during radiotracer injection could be used to measure LVEF for patients who undergo whole-body oncological (in case of cardiotoxic chemotherapy) or myocardial PET/CT examination.

LVEF can be assessed with cardiac-gated ^15^O-water PET/computed tomography (CT) [12–14]: ^15^O-water labels the blood pool, allowing long-acquisition and high-signal-to-noise ratio images at equilibrium, thus correlating well with the gold standard (MRI or planar ERNA) results. However, ^15^O-water must be produced by an on-site cyclotron, only available in research centers. Therefore, more recent studies showed that ECG-gated first-pass ^18^F-FDG PET/CT of the cardiac cavities may be an alternative to planar/SPECT ERNA for measuring LVEF [15]. However, the limited sensitivity of conventional analog PET/CT machines involves an acquisition time of several minutes, and ^18^F-FDG myocardial uptake may degrade the cavity myocardium contrast, thereby impacting the LVEF measurements. This problem may be avoided by using the most recent ultra-sensitive digital PET technologies, which yield images with acceptable signal-to-noise ratios with acquisition times of only a few seconds [16–19] and allow high counting rates without detector saturation [19], essential in first-pass acquisitions.

This feasibility study aims to evaluate whether ultra-short first-pass acquisition ^18^F-FDG digital PET/CT in the cardiac cavities accurately measures LVEF, using a 3D-printed pair of anthropomorphic heart.

## Methods

### Anthropomorphic heart phantom design

Two heart phantoms were printed from polylactic acid with the 3D printer Ultimaker 2+ (Ultimaker, Utrecht, the Netherlands) on the basis of artifact-free ECG-gated MR images of a patient. The ventricular cavity and myocardium contours in the phantoms corresponded to their shapes at end-diastole and end-systole. Thus, to obtain the phantom pair, the volumes were manually delineated with the Eclipse segmentation software (Varian Medical Systems, Palo Alto, USA), exported to the Slicer 3D software [20], converted into stereolithography (.STL) format, and imported into the 3D printer software (Ultimaker Cura). The phantoms were then 3D-printed. In this study, there is no additional material to simulate soft tissue attenuation, since we assume attenuation will impact all modalities in similar proportions.

### Determination of the reference LVEF value

The reference LVEF of the phantom pair was measured by weighing the two phantoms before and after filling their left ventricle with water (volumetric mass density 1 g/cm^3^). LVEF was calculated by using the following formula (1):

1 $$ LV\mathrm{EF}\ \left(\%\right)=\frac{\mathrm{EDV}-\mathrm{ESV}}{\mathrm{EDV}}\times 100 $$

where EDV is the left ventricle end-diastole volume, and ESV is the left ventricle end-systole volume.

### Imaging of the phantoms and data processing

**Fig. 1:**
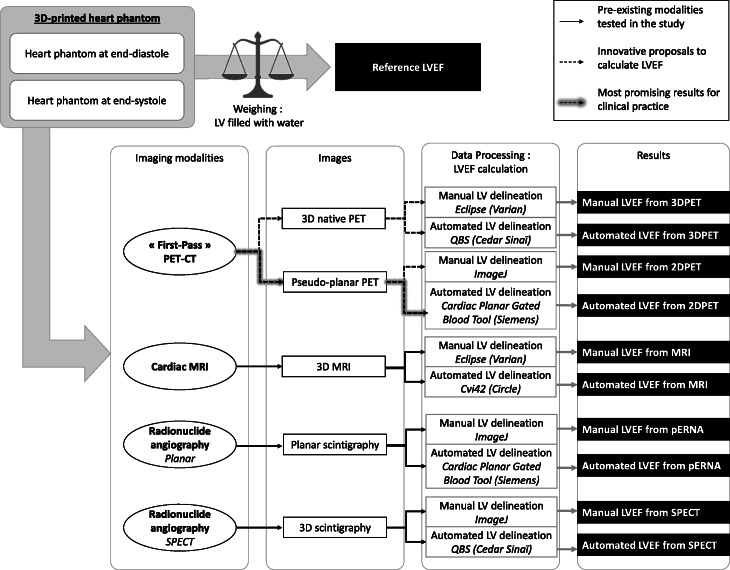
Schematic depiction of the protocol used to determine the LVEF in the heart-phantom pair. CT, computed tomography; LV, left ventricle; LVEF, left ventricular ejection fraction; MRI, magnetic resonance imaging; QBS, quantitative blood pool SPECT; PET, positron emission tomography; pERNA, planar equilibrium radionuclide angiocardiography; SPECT, single-photon emission computed tomography

The LVEF of the phantom pair was measured with cardiac MR imaging, planar and SPECT ERNA, and PET/CT. Table 1 summarizes the main acquisition conditions used with each modality to obtain heart cine-equivalent images and to compute LVEF. These conditions reproduce those of clinical acquisitions in terms of radiotracer concentration and acquisition duration so that the image quality closely resembles those of clinical images. For each modality, LVEF was calculated by post-processing the images with manual and automated segmentation methods. When possible, analyses were performed several times to check intra-observer variability (4 repetitions of analysis by the same observer) and inter-observer variability (4 repetitions from 4 different observers). Figure 1 summarizes the methodological plan of these studies.

**Table 1:** Acquisition conditions used with the four imaging modalities to measure LVEF in the heart-phantom pair

Imaging modality	Device	Radionuclide	Blood activity concentration	Acquisition time per phase	Automated clinical segmentation tool
**MRI**	Philips Ingenia 1.5T	–	–	≈ 10 min	Circle cvi42
**Planar ERNA**	Siemens Intevo Bold + LEHR collimator	^99m^Tc	110 kBq/mL	38 s	Siemens Gated Blood Pool tool
**SPECT ERNA**				5 s/projection 30 projections (every 3° angle)	Cedars Sinai Quantitative Blood Pool SPECT (QBS)
**PET/CT**	Siemens Biograph Vision 600	^18^F	210 kBq/mL	3 s	Cedars Sinai Quantitative Blood Pool (QBS) SPECT adapted to PET images

*ERNA* equilibrium radionuclide angiocardiography, *MRI* magnetic resonance imaging, *PET/CT* positron emission tomography/computed tomography, *SPECT* single-photon emission computed tomography

### MR imaging

MR images of the phantom pair were acquired on a Philips Ingenia 1.5T system (Philips Healthcare, Best, the Netherlands). The acquisition was processed by using a segmented K-space gradient-echo cardiac sequence [21, 22], with the left and right ventricular cavities filled with water. The MR images obtained had a 256 × 256-pixel matrix with 1.25 mm per pixel resolution and an 8-mm slice thickness. LVEF was manually calculated by transferring the images to the contouring module of Eclipse, delineating the left ventricle volumes, and then employing formula (1). LVEF was also automatically computed by a senior radiologist, using the Circle cvi42 software (Circle Cardiovascular Imaging Inc., Calgary, Canada).

### Planar and SPECT isotopic ventriculography

Isotopic ventriculographic images of the phantom pair were acquired on a Siemens Intevo Bold (Siemens Healthineers, Knoxville, USA) gamma-camera equipped with parallel holes low-energy high-resolution (LEHR) collimator. We determined how much radionuclide activity to add to each phantom on the basis of the ^99m^Tc blood concentration present in the average patient during isotopic ventriculographic examinations. Thus, the average 70-kg adult is injected with 740 MBq of ^99m^Tc pertechnetate. Of this, as little as 75% of the ^99m^Tc pertechnetate is taken up by the erythrocytes: this reflects the fact that although 95% of the ^99m^Tc pertechnetate is generally taken up by the erythrocytes [23], it remains possible that the erythrocyte surface uptake is reduced, in which case the labeling drops by 20% [24]. Thus, the minimum effective activity in the blood of the average patient during isotopic ventriculography would be 555 MBq. At the moment of the acquisition (several minutes after injection), this activity would be homogeneously diluted in the patient’s blood pool, which corresponds to approximately 5 L. Thus, the ^99m^Tc concentration in the blood in the cardiac cavities would be approximately 110 kBq/mL.

Planar images were acquired with oblique anterior incidence to separate the left and right ventricles. In clinical conditions, planar images are acquired over 600 s (approximately 400,000 counts per image, recommended by the French Society of Nuclear Medicine [25]), and a 16-gate cine image is produced by retrospective reconstruction according to the ECG signal; thus, each gate is set at 37.5 s. Therefore, the planar ERNA acquisition time for each phantom was set to 38 s. LVEF was calculated manually by processing the planar images with the ImageJ software [26] and then counting the events in the left ventricle areas. LVEF was also calculated automatically from the planar images on the basis of the event counts in the left ventricle areas by processing the images with the Cardiac Planar Gated Blood Tool (Siemens Healthineers, Erlangen, Germany).

Gated SPECT images were acquired with an acquisition time of 5 s/projection per frame (for each phantom), which corresponds to 80 s/projection in clinical conditions. We made 32 projections covering a 180° angle with detectors positioned in “L-mode” (90°) and following a circular orbit centered on the phantom. All SPECT images had a 64 × 64-pixel matrix with 4.2 mm per pixel resolution (zoom 2) and a 4.2-mm slice thickness. LVEF was calculated manually by processing the SPECT images with the ImageJ software delineating the ventricle volumes and using formula (1). LVEF was also calculated from the SPECT images by automatically segmenting the left ventricle in the images with Quantitative Blood Pool SPECT (QBS), a validated clinical tool (Cedars-Sinai Medical Center, Los Angeles, USA) [27].

### PET/CT images

PET/CT images were acquired with a digital Siemens Biograph Vision 600 PET/CT system (Siemens Healthineers, Knoxville, USA). We determined how much radionuclide activity to add to each phantom on the basis of the average ^18^F-FDG concentration in the blood a few seconds after the bolus injection: to image the first-pass of the tracer into the heart cavities in clinical conditions, acquisitions are started as soon as the bolus is injected. The average ^18^F-FDG concentration was calculated on the basis of two assumptions. First, the average 70-kg adult is injected with 140 MBq of ^18^F-FDG (although we have observed in our clinical practice that 2 MBq/kg of ^18^F-FDG is sufficient to obtain good-quality images with this PET/CT system). Second, when the image is acquired during the bolus injection (first-pass image), the injected activity is diluted in a small blood volume: assuming an average cardiac flow of 5 L/min, this volume is approximately 670 mL. This is the blood volume ejected by the heart during the first heartbeats (approximately 8 s), which is the time needed to acquire an image. Thus, the ^18^F-FDG activity in the cardiac cavities was estimated to be approximately 210 kBq/mL.

We assume an 8-phase segmentation of the clinical cine acquisition of the cardiac cycle to ensure a good enough signal-to-noise ratio in this very short 8-s acquisition. Consequently, each phase would correspond to an acquisition duration of 1 s. However, the shortest reconstruction frame duration allowed by the Biograph Vision PET console is 3 s. Therefore, we acquired the heart signals from phantoms over 3 s.

The 3D PET images were reconstructed with an ordered subset expectation maximization (OSEM) 3D iterative algorithm (4 iterations, 5 subsets) with all corrections (normalization, dead time, attenuation, decay, scattering, and random events, estimated by delayed window technique), time-of-flight and point spread function. As with clinical reconstructions, 2.2-mm FWHM Gaussian post-filtering was applied to optimize the contrast-to-noise ratio in the reconstructed images which had a 220 × 220-pixel matrix with 1.65 mm per pixel resolution in the axial plane and a 1.65-mm slice thickness. For automatic QBS post-processing, the end-systole and end-diastole images of the phantom were co-registered in the X, Y, and Z directions, gathered, converted into Nuclear Medicine-type images with a homemade Python tool, and saved in the DICOM (“NM” modality) format. The 3D images were reoriented according to the cardiac small axis and cropped from 220 × 220 × 220 to 128 × 128 × 128 while maintaining voxel size. Each reoriented volume was reproduced another three times, and then all four reproductions were included into a wide final volume, thus producing 4 end-diastole volumes and 4 end-systole volumes (8 phases). The DICOM header was built on the model of an 8-phase gated-SPECT (“NM” modality format). LVEF was calculated by employing the 8-phase gated nuclear medicine images as input files in QBS and delineating the left ventricle in an automated fashion, using surface-, count-, and volume-based methods. The native PET images were also exported to the contouring module of Eclipse to delineate manually left ventricle and calculate LVEF using formula (1).

**Fig. 2:**
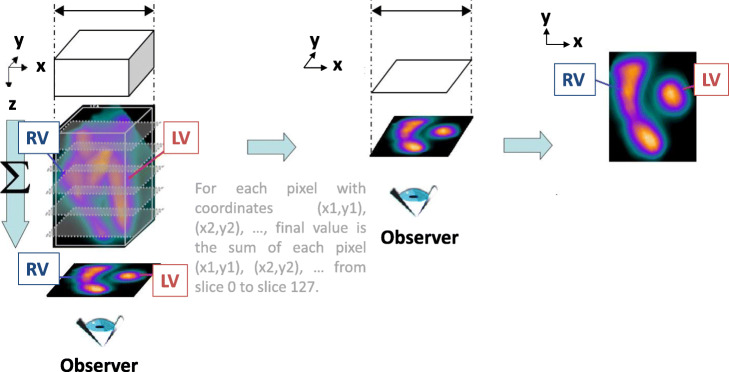
Schematic depiction of the automated Python method for producing “pseudo-planar” PET images. The “pseudo-planar” image was obtained by summing the signals in all 127 PET slices along the *z*-axis. Preorientation allowed the *z*-axis to be aligned with the cardiac small axis. LV, left ventricle; RV, right ventricle

In addition, the native 3D images were summed along the cardiac short axis and horizontal long axis to produce 2D “pseudo-planar” images that were equivalent to those obtained with planar ERNA. This summation was conducted manually with ImageJ (Z-project) or in an automated fashion with our homemade Python tool. The latter is illustrated in Fig. 2, which shows the 3D array of one cardiac phase oriented along the cardiac small axis: the pixels in all 127 slices at the same coordinates (*x*,*y*) are summed, generating a unique value for each coordinate (*x*,*y*). This summation operation is performed for the short-axis and horizontal long-axis directions. Thus, this process led to eight 2D acquisitions (one for each phase) to mimic a clinical 8-phase planar ERNA image, with a DICOM header built on the model of an 8-phase gated planar acquisition. The short-axis and horizontal long-axis 2D “pseudo-planar” images were used to calculate LVEF by counting all events in the left-ventricle region of interest (as with planar ERNA). Both manual (ImageJ) and automated (QBS) segmentations were used.

## Results

### Anthropomorphic heart phantom

**Fig. 3:**
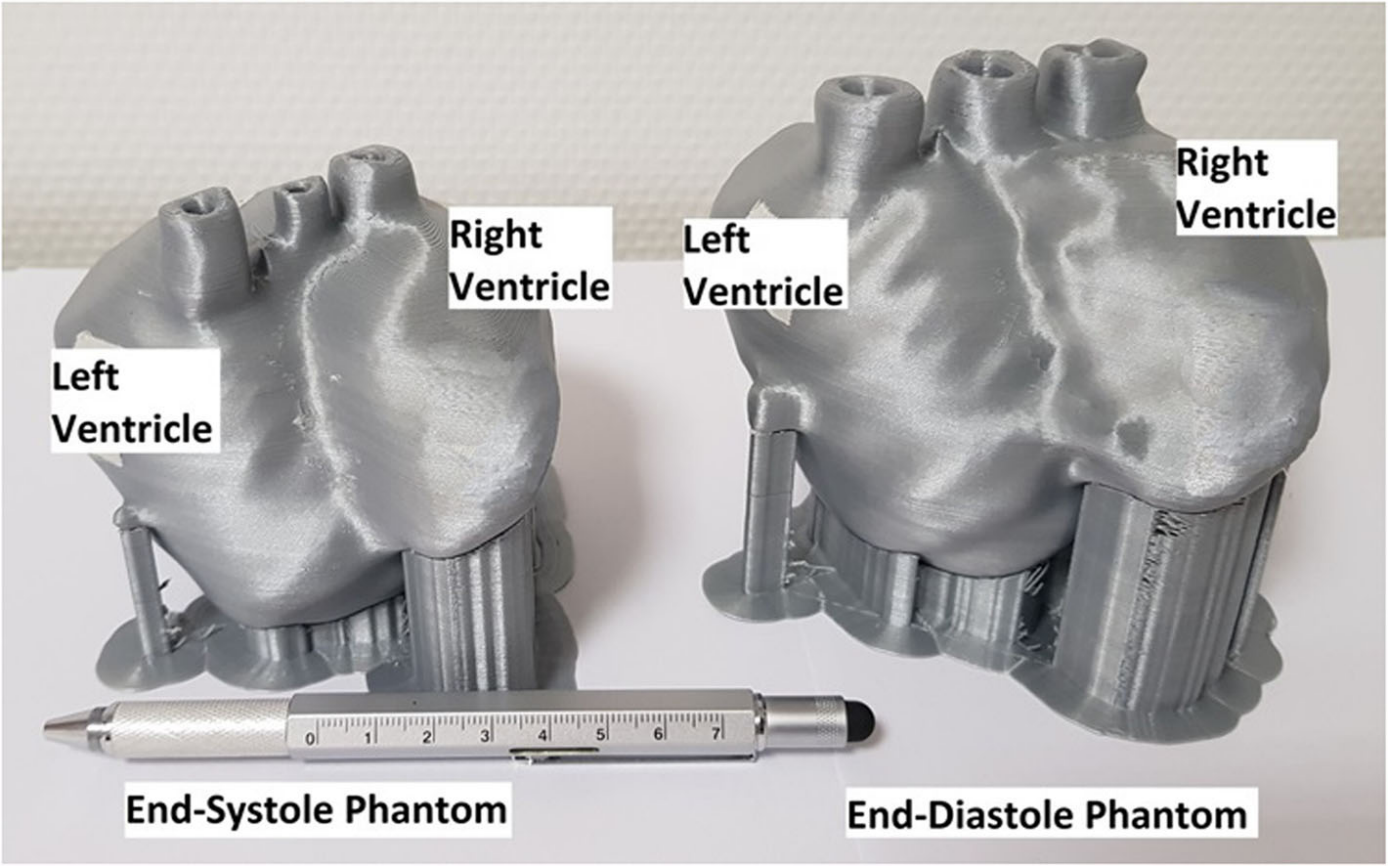
The 3D-printed heart-phantom pair showing the heart at end-diastole and end-systole. The pipes on the top of the phantoms were used to fill the left and right cavities and the myocardium compartment with water or radionuclide solutions

The 3D-printed phantom pair is shown in Fig. 3.

### Reference LVEF value

The left ventricular end-diastole volume (EDV) and end-systole volume (ESV) reference values were 110 and 62 cm^3^, respectively. They were used with formula (1) to calculate the reference LVEF, which was 43.6% (Table 2).

**Table 2:** Left ventricle end-diastole and end-systole volumes and ejection fraction of the heart-phantom pair. These values were measured with four imaging modalities and calculated with manual and automated methods

Modality	Manual EDV, cm^**3**^ (m±SD)	Automated EDV, cm^**3**^ (m±SD)	Manual ESV, cm^**3**^ (m±SD)	Automated ESV, cm^**3**^ (m±SD)	Manual LVEF, % (m±SD)	Automated LVEF, % (m±SD)
**Reference value**	110	–	62	–	***43.6***	–
**MRI**	107.0 ± 6.4 *108.8* ± *3.2*	120	59.7 ± 4.8 *59.5* ± *2.4*	67	44.3 ± 1.7 *45.3* ± *1.0*	44.7
**SPECT ERNA**	145.7 ± 50.2 *111.8* ± *8.8*	113^1^ 120.8 ± 3.4^2^	89.1 ± 41.2 *75.3* ± *13.5*	A.1.1.1.1.1.1. 60^1^ 51.8 ± 12.4^2^	40.3 ± 12.2 *32.8* ± *10.0*	A.1.1.1.1.1.2. 46.9^1^ 57.1 ± 10.3^2^
**Planar ERNA**	–	–	–	–	46.0 ± 2.8 *45.1* ± *6.7*	46.4
**Native 3D PET/CT**	103.9 ± 4.9 *108.5* ± *4.9*	89.5 ± 0.6^3^	54.6 ± 2.6 *58.4* ± *2.5*	48.0 ± 0^3^	47.5 ± 1.9 *46.2* ± *1.6*	46.4 ± 0.3^3^
**“Pseudo-planar” short-axis PET**	–	–	–	–	**43.6** ± 0.7 *44.2* ± *0.1*	***43.6***
**“Pseudo-planar” horizontal long-axis PET**	–	–	–	–	45.5 ± 0.7 *44.6* ± *0.5*	–

For manual and semi-automated processes, the value corresponds to the average of 4 repetitions of the image treatment ± the standard deviation. Roman characters indicate results over 4 repetitions with 4 different observers (inter-observer variability) whereas italic characters indicate results over 4 repetitions with the same observer (intra-observer variability)

*CT* computed tomography, *EDV* left ventricle end-diastole volume, *ERNA* equilibrium radionuclide angiocardiography, *ESV* left ventricle end-systole volume, *LVEF* left ventricular ejection fraction, *MRI* magnetic resonance imaging, *PET* positron emission tomography, *SPECT* single-photon emission tomography

^1^These SPECT values were calculated in a full automated fashion by using the QBS surface-based method

^2^These SPECT values were calculated in a semi-automated fashion by using the QBS surface-based method after manually placement of the anatomical landmarks—valves and septum—to help segmentation

^3^These PET/CT-derived values were calculated in a semi-automated fashion by using the QBS surface-based method after manually defining the anatomical landmarks—valves and septum—to help segmentation. Full automated QBS computation of PET images failed to identify the left ventricle volumes

### LVEF measurements using the heart-phantom pair with gold standard imaging modalities

**Fig. 4:**
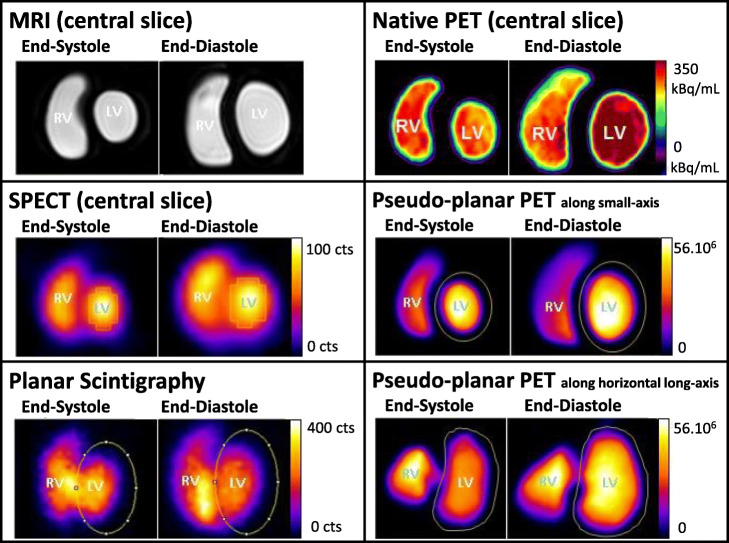
Images of the end-diastole/end-systole heart-phantom pair. For the 3D modalities, namely, magnetic resonance imaging (MRI), single-photon emission computed tomography (SPECT), and native positron emission tomography (PET), only the central slice in the cardiac short axis is shown. The SPECT, planar scintigraphy, and “pseudo-planar” PET images show representative examples of manual segmentation. The “pseudo-planar” PET images along the cardiac small axis had better image quality (especially in terms of spatial resolution) than the equivalent planar scintigraphy images. LV, left ventricle; RV, right ventricle

**Fig. 5:**
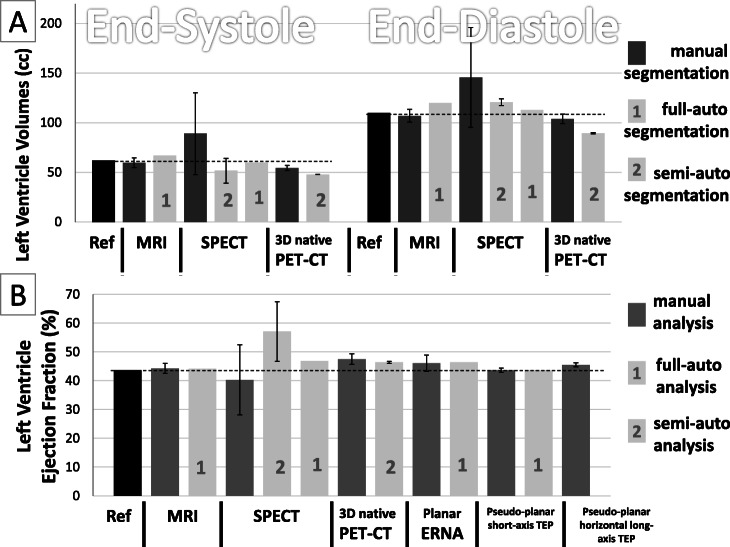
Left ventricle **A** volumes and **B** ejection fractions of the heart-phantom pair. These values were measured by MRI, SPECT, planar scintigraphy, native 3D PET/CT, and “pseudo-planar” PET/CT projections on the cardiac small axis and horizontal long axis. LVEF, left ventricular ejection fraction. The prefixes “a” and “m” signify automated and manual calculations, respectively. Left ventricle end-diastole and end-systole volumes are not shown in **A** for planar scintigraphy or the “pseudo-planar” PET/CT projections because these modalities do not provide information about left ventricle volumes. The automated left ventricle volumes and LVEF values shown for SPECT and native PET were calculated by using the surface-based QBS method. ^1^“Full automated” computations represent analysis with no human intervention, so no variability expected nor observed. ^2^“Semi-automated” processes stand for automated computation of LVEF after a manual placement of anatomical landmarks (valves and septum). For semi-automated and manual processes, the value corresponds to the average of 4 repetitions of the image treatment with 4 different operators, and the error bar represents the standard deviation (inter-operator variability)

The heart phantom images obtained with MR imaging and planar and SPECT ERNA are presented in Fig. 4. The EDV, ESV, and LVEF, calculated by manual and automated methods, are summarized in Table 2 and Fig. 5. All EDVs, ESVs, and LVEFs measured with manual and automated methods are close to the reference values, except for manual delineation of SPECT images that over-estimates the left ventricle volumes and presents an important inter-observer variability. SPECT-automated delineation with QBS also gives more accurate and reproducible results when used in full automated fashion without any human intervention to place anatomical landmarks.

### LVEF measurement using the heart-phantom pair with PET/CT

The heart phantom images that were obtained with native 3D PET/CT and “pseudo-planar” PET/CT are presented in Fig. 4.

Regarding native PET/CT, average manual (Eclipse) calculations yielded slightly underestimated EDV and ESV compared to the reference values. However, this method provided an average LVEF close to the reference value, with good intra- and inter-observer reproducibility over the left ventricle volumes and LVEF.

By contrast, automated (QBS) calculations that were based on surface, volume, and counts had heterogeneous results: the respective EDVs were 89.5, 100, and 100 cm^3^; the respective ESVs were 48, 53, and 32 cm^3^; and the respective LVEFs were 46.4%, 47.0%, and 68.0%. Note that in Table 2 and Fig. 5, we only showed the surface-based calculations of EDV, ESV, and LVEF. From “pseudo-planar” PET images summed along the short axis, the LVEF values, calculated directly from the left ventricle event counts with manual (ImageJ) and automated (Cardiac Planar Gated Blood Tool) methods, were close/identical to the reference LVEF. “Pseudo-planar” PET images summed along the horizontal long axis provided similar results on manual (ImageJ) analysis: the LVEF was also close to the reference LVEF. An automated tool for calculating LVEF from these images does not exist.

## Discussion

### 3D image analyses

**Fig. 6:**
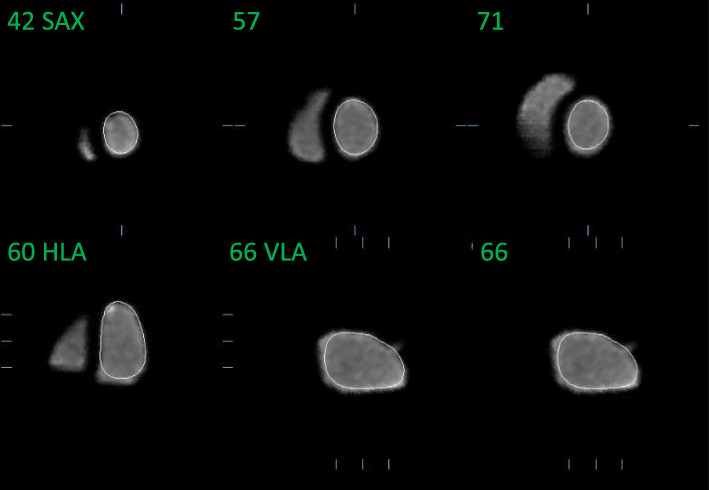
Automated segmentation with quantitative blood pool SPECT (QBS) of native 3D PET/CT images at end-diastole. The lines indicate the imprecise ventricle edges that were determined by this automated segmentation algorithm

The reference EDV, ESV, and LVEF were 110 cm^3^, 62 cm^3^, and 43.6%, respectively. Manual data processing of the MR and 3D PET/CT images yielded similar EDVs (103.9–107 cm^3^), ESVs (54.6–59.7 cm^3^), and LVEFs (44.3–47.5%). Manual data processing of the SPECT images yielded less accurate and less reproducible EDVs (89.1 ±41.2 cm^3^), ESVs (145.7 ± 50.2 cm^3^), and LVEF (40.3 ± 12.2), due to the poor sampling matrix of SPECT images (pixel size = 6.6 mm) and the difficulty of producing precise delineations on 3D images with non-dedicated software ImageJ. Processing the MR and SPECT images with full automated clinical tools also yielded good estimations of EDV and ESV (113–120 and 60–67 cm^3^, respectively) and LVEF (44.2% and 46.9%, respectively). By contrast, full automated processing of the 3D PET images with the QBS tool did not converge to any left ventricle delineation. When helped by manual placement of the valves and septum, it markedly underestimated the ventricular volumes: use of the surface-, volume-, and count-based methods in QBS respectively led to EDV values of 89.5, 100, and 100 cm^3^ (versus the 110 cm^3^ reference value) and ESV values of 48, 53, and 32 cm^3^ (versus the 62 cm^3^ reference value). As a result, the LVEF after automated 3D PET image processing ranged widely from 46.4 to 68.0%. Although the surface-based QBS method yielded an LVEF that was quite close to the reference LVEF (46.7% versus 43.6%), the inconsistent ventricular volume values produced by this automated method cast doubt on the accuracy with which it generates LVEF values. These observations suggest that QBS, originally developed for SPECT images, is not suitable for 3D PET images. This may reflect the excellent spatial resolution of the PET images: by contrast, SPECT images have a much lower spatial resolution (around 1 cm in SPECT versus <4 mm with our PET system). It is accommodated in the QBS segmentation algorithm, which acts by generating ellipsoids [27, 28]. Consequently, QBS may not be able to manage the PET spatial resolution, causing small structures such as the apex to be excluded during segmentation. Indeed, this phenomenon is visible in Fig. 6. Another possible source of QBS error may have been the simplistic structure of our phantom that lacked key anatomical structures such as the heart atria or the valvular planes, making it difficult for the algorithm to place anatomical landmarks during segmentation.

Thus, at present, native 3D PET images can only be used to calculate LVEF with a slice-by-slice manual segmentation method.

### “Pseudo-planar” PET images

While manual segmentation of the left ventricle in each slice of the 3D PET-CT images produced accurate results, it was a very time-consuming process. Therefore, we also sought to produce “pseudo-planar” PET images similar to those acquired by planar ERNA. These images were easy to process manually since only single regions of interest had to be drawn around the end-diastole and end-systole left ventricles; the event counts in these regions were then used to calculate the LVEF. This process is shown in Fig. 4.

**Fig. 7:**
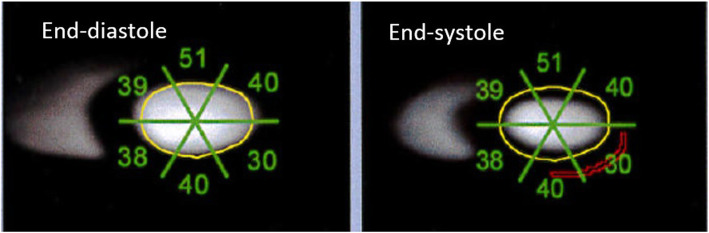
Automated segmentation of “pseudo-planar” PET/CT images summed along the cardiac short axis

We found that when the “pseudo-planar” PET images were processed manually (ImageJ) or in an automated fashion with the clinical Cardiac Planar Gated Blood Tool, the LVEF was very close to the reference LVEF (43.6–45.5% versus 43.6%). In fact, automated segmentation on the short-axis projections yielded exactly the same LVEF as the reference value (Fig. 7). Thus, automated segmentation of “pseudo-planar” short-axis projections with the Cardiac Planar Gated Blood Tool seems to be the most promising PET/CT approach because it assures accurate, fast, and reproducible LVEF estimations.

### Study limitations

The main study limitation is that the phantom pair is motionless and thus cannot be used to determine the effect of temporal sampling on LVEF and ventricular volume measurements. In our institution, the cardiac cycle is divided into 30 frames in MR imaging, 16 frames in radionuclide angiography, and 8 frames in the present PET/CT study. Whether temporal sampling can impact these measurements remains to be determined.

All imaging modalities yielded strongly contrasted images of the phantom; such contrast is generally not observed for real anatomical structures. However, the lack of myocardium signals on the PET imaging analyses may be representative of the clinical situation a few seconds after radiotracer injection because, at this point, the ^18^F-FDG bolus will not have been taken up by the myocardial wall. Indeed, because of this, we deliberately filled the myocardial compartment with non-radioactive water for PET/CT imaging.

Another drawback of our study may be that our phantom pair was missing anatomical structures (atria and valves). It is possible that the absence of these structures hampered the automated segmentation of the 3D PET images of the left ventricle by the QBS clinical software. A more elaborate beating heart phantom may allow QBS to measure LVEF more accurately from 3D PET images. Additional studies with such phantoms are warranted.

## Conclusions

To our knowledge, this is the first study to evaluate whether LVEF can be accurately measured by first-pass PET images acquired by ultra-sensitive digital PET systems. Our study with an anthropomorphic heart-phantom pair showed that such a PET system generated high-quality images over a very fast acquisition duration of only 3 s. We also showed that manual processing of the 3D PET images yielded a good LVEF value whereas automated processing yielded less trustworthy LVEF values. By contrast, when summed 2D “pseudo-planar” PET images on the cardiac short axis were generated, automated processing yielded even better LVEF estimates than manual processing. Thus, converting 3D PET/CT images to “pseudo-planar” PET images on the cardiac short axis and then segmenting the images with an automated approach using Cardiac Planar Gated Blood Tool may be the easiest and most efficient method for determining LVEF with PET/CT in clinical practice. This approach may be useful for concomitantly measuring LVEF in patients undergoing PET/CT, thus side-stepping the limitations of the gold standard methods that are currently used to measure LVEF. These encouraging results will be confirmed in patients.

## Data Availability

The datasets used and/or analyzed during the current study are available from the corresponding author on reasonable request.

## Abbreviations

ECG: Electrocardiogram
EDV: Left ventricle end-diastolic volume
ERNA: Equilibrium radionuclide angiocardiography
ESV: Left ventricle end-systolic volume
^18^F-FDG: ^18^F-fluorodeoxyglucose
LVEF: Left ventricular ejection fraction
MR: Magnetic resonance
PET/CT: Positron emission tomography/computed tomography
QBS: Quantitative blood pool SPECT
SPECT: Single-photon emission computed tomography
^99m^Tc: Technetium^99m^

## References

[1] Marcu CB, Beek AM, van Rossum AC (2006). Clinical applications of cardiovascular magnetic resonance imaging. CMAJ.

[2] Wackers FJT, Berger HJ, Johnstone DE, Goldman L, Reduto LA, Langou RA (1979). Multiple gated cardiac blood pool imaging for left ventricular ejection fraction: validation of the technique and assessment of variability. Am J Cardiol.

[3] Nichols KJ, Tosh AV, Wang Y, Palestro CJ, Reichek N (2009). Validation of gated blood-pool SPECT regional left ventricular function measurements. J Nucl Med.

[4] Berning J, Nielsen JR, Launbjerg J, Fogh J, Mickley H, Andersen PE (1992). Rapid estimation of left ventricular ejection fraction in acute myocardial infarction by echocardiographic wall motion analysis. CRD..

[5] Johri AM, Picard MH, Newell J, Marshall JE, King MEE, Hung J (2011). Can a teaching intervention reduce interobserver variability in LVEF assessment: a quality control exercise in the echocardiography lab. JACC Cardiovasc Imaging.

[6] Lum DP, Coel MN (2003). Comparison of automatic quantification software for the measurement of ventricular volume and ejection fraction in gated myocardial perfusion SPECT. Nucl Med Commun.

[7] Zuo-Xiang H, Cwaig E, Preslar JS, Mahmarian JJ, Verani MS (1999). Accuracy of left ventricular ejection fraction determined by gated myocardial perfusion SPECT with Tl-201 and Tc-99m sestamibi: comparison with first-pass radionuclide angiography. J Nucl Cardiol.

[8] Foley TA, Mankad SV, Anavekar NS, Bonnichsen CR, Morris MF, Miller TD (2012). Measuring left ventricular ejection fraction-techniques and potential pitfalls. Eur Cardiol.

[9] Gopal AS, Shen Z, Sapin PM, Keller AM, Schnellbaecher MJ, Leibowitz DW (1995). Assessment of cardiac function by three-dimensional echocardiography compared with conventional noninvasive methods. Circulation.

[10] Persson E, Carlsson M, Palmer J, Pahlm O, Arheden H (2005). Evaluation of left ventricular volumes and ejection fraction by automated gated myocardial SPECT versus cardiovascular magnetic resonance. Clin Physiol Funct Imaging..

[11] Friedman JD, Berman DS, Borges-Neto S, Hayes SW, Johnson LL, Nichols KJ, Pagnanelli RA, Port SC, Quality Assurance Committee of the American Society of Nuclear Cardiology (2006). First-pass radionuclide angiography. J Nucl Cardiol. nov.

[12] Nordström J, Kero T, Harms HJ, Widström C, Flachskampf FA, Sörensen J (2017). Calculation of left ventricular volumes and ejection fraction from dynamic cardiac-gated 15O-water PET/CT: 5D-PET. EJNMMI Phys.

[13] Ben Bouallègue F, Mariano-Goulart D, Agostini D, Manrique A (2018). Feasibility of biventricular volume and function assessment using first-pass gated 15O-water PET. EJNMMI Res.

[14] Driessen RS, van Timmeren JE, Stuijfzand WJ, Rijnierse MT, Danad I, Raijmakers PG, Beek AM, van Rossum AC, Nijveldt R, Lammertsma AA, Harms HJ, Huisman MC, Knaapen P (2016). Measurement of LV volumes and function using oxygen-15 water-gated PET and comparison with CMR imaging. J Am Coll Cardiol Cardiovasc Imaging.

[15] Ben Bouallègue F, Maïmoun L, Kucharczak F, Le Fur P, Vauchot F, Hay B, et al. Left ventricle function assessment using gated first-pass 18F-FDG PET: validation against equilibrium radionuclide angiography. J Nucl Cardiol. 2021;28(2):594-603. 10.1007/s12350-019-01731-x 31044403

[16] Lecomte R (2009). Novel detector technology for clinical PET. Eur J Nucl Med Mol Imaging.

[17] Wagatsuma K, Miwa K, Sakata M, Oda K, Ono H, Kameyama M, Toyohara J, Ishii K (2017). Comparison between new-generation SiPM-based and conventional PMT-based TOF-PET/CT. Phys Med.

[18] Del Guerra A, Belcari N, Giuseppina M, LLosa G, Marcatili S, Ambrosi G, et al. Advantages and pitfalls of the silicon photomultiplier (SiPM) as photodetector for the next generation of PET scanners. Nuclear Instruments and Methods in Physics Research Section A: Accelerators, Spectrometers, Detectors and AssociatedbEquipment. 2010;617:223–6.

[19] van Sluis JJ, de Jong J, Schaar J, Noordzij W, van Snick P, Dierckx R, et al. Performance characteristics of the digital Biograph Vision PET/CT system. ​J Nucl Med. 2019;60(7):1031–6. 10.2967/jnumed.118.215418 30630944

[20] Kikinis R, Pieper SD, Vosburgh KG. 3D Slicer: a platform for subject-specific image analysis, visualization, and clinical support. In: Jolesz FA, éditeur. Intraoperative imaging and image-guided therapy [Internet]. New York: Springer; 2014 p. 277-289. [cité 17 avr 2020]. Disponible sur: 10.1007/978-1-4614-7657-3_19.

[21] Atkinson DJ, Edelman RR (1991). Cineangiography of the heart in a single breath hold with a segmented turboFLASH sequence. Radiology.

[22] Bluemke DA, Boxerman JL, Atalar E, McVeigh ER (1997). AJR Am J Roentgenol.

[23] Pavel DG, Zimmer M, Patterson VN (1977). In vivo labeling of red blood cells with 99mTc: a new approach to blood pool visualization. J Nucl Med.

[24] Technescan PYP - Summary of product characteristics [Internet]. [cité 1 mai 2020]. Disponible sur: http://agence-prd.ansm.sante.fr/php/ecodex/rcp/R0207613.htm.

[25] Guide pour la rédaction de protocoles pour la ventriculographie isotopique à l’équilibre. :23.

[26] Schneider CA, Rasband WS, Eliceiri KW (2012). NIH Image to ImageJ: 25 years of image analysis. Nat Methods.

[27] Harel F, Finnerty V, Grégoire J, Thibault B, Marcotte F, Ugolini P, Khairy P (2010). Gated blood-pool SPECT versus cardiac magnetic resonance imaging for the assessment of left ventricular volumes and ejection fraction. J Nucl Cardiol.

[28] Van Kriekinge SD, Berman DS, Germano G (1999). Automatic quantification of left ventricular ejection fraction from gated blood pool SPECT. J Nucl Cardiol.

